# Mineralogical characteristics of sediments and heavy metal mobilization along a river watershed affected by acid mine drainage

**DOI:** 10.1371/journal.pone.0190010

**Published:** 2018-01-05

**Authors:** Yingying Xie, Guining Lu, Chengfang Yang, Lu Qu, Meiqin Chen, Chuling Guo, Zhi Dang

**Affiliations:** 1 School of Environment and Energy, South China University of Technology, Guangzhou, China; 2 The Key Laboratory of Pollution Control and Ecosystem Restoration in Industry Clusters, Ministry of Education, South China University of Technology, Guangzhou, China; 3 Guangdong Provincial Engineering and Technology Research Center for Environmental Risk Prevention and Emergency Disposal, South China University of Technology, Guangzhou, China; 4 School of Environmental and Biological Engineering, Guangdong University of Petrochemical Technology, Maoming, China; Sun Yat-Sen University, CHINA

## Abstract

Trace-element concentrations in acid mine drainage (AMD) are primarily controlled by the mineralogy at the sediment-water interface. Results are presented for a combined geochemical and mineralogical survey of Dabaoshan Mine, South China. Developed sequential extraction experiments with the analysis of the main mineralogical phases by semi-quantitative XRD, differential X-ray diffraction (DXRD) and scanning electron microscopy (SEM) were conducted to identify the quantitative relationship between iron minerals and heavy metals. Results showed that schwertmannite, jarosite, goethite and ferrihydrite were the dominant Fe-oxyhydroxide minerals which were detected alternately in the surface sediment with the increasing pH from 2.50 to 6.93 along the Hengshi River. Decreasing contents of schwertmannite ranging from 35 wt % to 6.5 wt % were detected along the Hengshi River, which was corresponding to the decreasing metal contents. The easily reducible fractions exert higher affinity of metals while compared with reducible and relatively stable minerals. A qualitative analysis of heavy metals extracted from the sediments indicated that the retention ability varied: Pb > Mn > Zn > As ≈ Cu > Cr > Cd ≈ Ni. Results in this study are avail for understanding the fate and transport of heavy metals associated with iron minerals and establishing the remediation strategies of AMD systems.

## Introduction

Acid mine drainage (AMD), with elevated concentrations of SO_4_^2-^ and metal cations such as Fe^3+^, is triggered by the exposure of atmospheric water and oxygen of sulfide minerals [1]. And it comprises a concatenated series of mineralogical and chemical reactions between sediments and AMD water [1]. Researches on the ochreous precipitates from AMD have shown that the deposits contain a diverse group of iron oxy-hydroxide minerals, in particular, the poorly ordered hydroxysulfates (e.g., jarosite and schwertmannite) that are powerful sorbents for trace metals and oxyanions [2]. Additionally, the release of heavy metals into the groundwater is associated with Fe(III) oxyhydroxide minerals by dissolution or transformation [3–7]. Due to the potential ecological and human health risks [8–9] associated with such a release, the growing awareness of the environmental protection calls for a better understanding of the mineralogical characteristics and transport of metals under extremely acidic conditions of AMD. Thus, a sound geochemical investigation is required at all stages to evolve the effective remediation of AMD area [10–11].

Based on the previous reports [12–14], during or after formation of iron oxy-hydroxide minerals, heavy metals can also be adsorbed onto minerals surfaces or trapped in structural regions in minerals. In AMD contaminated rivers, sediments consist of a complex mixture of secondary iron phases containing potentially toxic metal elements [15]. For example, Regenspurg and Peiffer [16] reported that the high concentrations of Cr (up to 812 mg/kg) and As (up to 6740 mg/kg) were detected in precipitates of the mineral schwertmannite in AMD affected areas. As reported by Carlson et al. [12] in AMD system in Finland, ochreous precipitates containing 5500–69800 mg/kg As were composed of schwertmannite, ferrihydrite, and goethite. Mean concentrations of As, Cd, Cu, Pb and Zn were measured to be 1580, 9.0, 160, 710 and 1730 mg/kg in sediments collected along the Daduk creek, Korea [17]. Hence, investigations on the mineralogy of precipitates which linked to critical environmental processes including trace element cycling in AMD area were ugly needed [5].

According to the literature, attention has been paid to qualitative analysis of iron oxy-hydroxide minerals or heavy metals by sequential extractions [18–19]. The existing sequential extraction procedures used to characterize the partitioning of metals in contaminated sediments were reported to be chemical extractions like BCR [20] and Tessier et al. [21]. The modified procedure distinguishes between the fractions that are exchangeable, specifically adsorbed, bound to oxyhydroxides, organic fraction and residual [22–23]. In order to distinguish the poorly crystalline iron minerals and crystalline iron oxides, ammonium oxalate or NH_4_-acetate was applied to separate the poorly order oxyhydroxides from insoluble highly ordered oxyhydroxides in sediments [1, 24]. However, the current extraction procedures would be inadequate for specific sediments from AMD system, owing to the nonspecificity of extractants and re-adsorption of trace metals into other phase minerals [23, 25]. With respect to this problem, attempts will make to improve sequential extractions towards higher selectivity and higher operational efficiency. In addition, differential X-ray diffraction (DXRD) combined with semi-quantitative XRD will applied as qualitative method for qualitative analysis of poorly order oxyhydroxides. Therefore, in the current study, special focus will be given to a developed sequential extraction methodology combined with DXRD and semi-quantitative XRD for quantifying proportions of minerals in Dabaoshan Mine, South China.

Although there has been a great deal of research interest paid to the study of fractional distribution, behavior and risk assessment of heavy metals in Dabaoshan Mine [26–28], however, less attention has been given to the mineralogical characteristics of sediments and their roles in heavy metals retention associated with AMD systems in Dabaoshan Mine. Here we evaluate a sequential extraction procedure designed for Dabaoshan Mine in terms of DXRD and semi-quantitative XRD, and elucidate the relationship between minerals and heavy metals comprehensively. Hence, the objectives of the present study are 1) to gain insight into the mineralogical characteristics of sediments in AMD-affected area, and 2) to understand the transport and fates of the metals. The information resulting from this study will help provide theoretical foundation for remediation strategies.

## Experimental

### Sampling and pre-treatment

**Fig 1:**
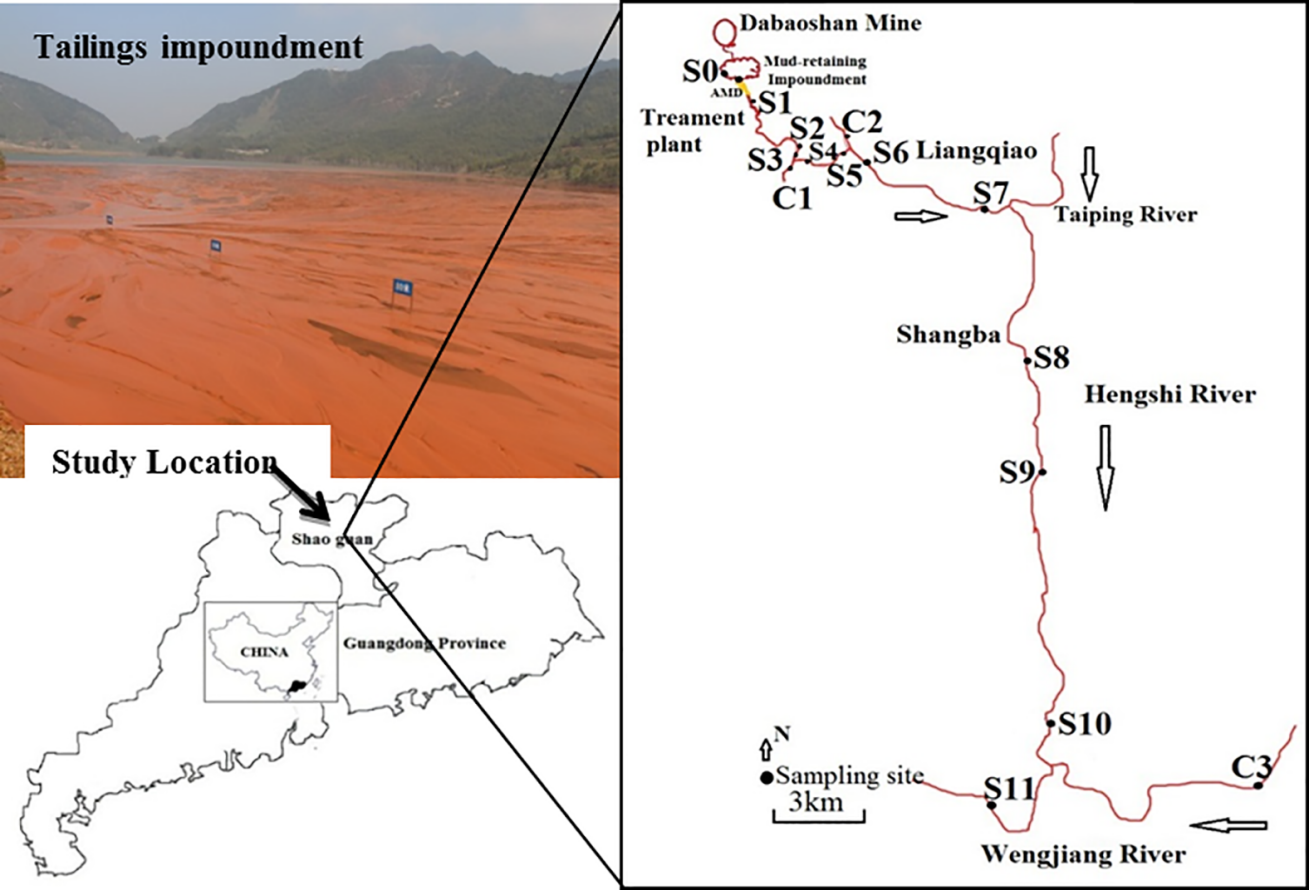
Map showing the locations of studied area of Dabaoshan Mine. (● represented the sample sites; S0 was tailings impoundment; the S1 to S11 located in the Hengshi River affected by AMD; C1 to C3 were control sites located in the tributaries).

The Dabaoshan Mine, a sulfide-bearing type of polymetallic deposit is located in the northern Guangdong Province (24°31′37″N; 113°42′49″E), southern China. As with most metal deposits and metal sulphide ores such as pyrite (FeS_2_), it is easily to be subject to rapid oxidation by weathering. Thus, AMD from the Dabaoshan Mine has caused severe acidification in the receiving stream, Hengshi River. Sediment and water samples were collected from Dabaoshan Mine (The sampling site was shown in Fig 1, including S0 to S11 (affected sites) and C1 to C3 (control sites)), along the Hengshi River in November, 2014. Sediment samples were taken from the surface sediments (1-2cm), in water depths of 0.5 m. At each site, one plastic core tube (70 mm in diameter) was manually pushed into the sediment to collect the samples. All the samples including water and sediment samples were kept in the refrigerator at 4°C prior to chemical analysis. pH values were measured in the field using a multi-parameter tester (SG2-T SevenGo). Water subsamples for metals, and anions were filtered through 0.45μm filters. All the samples used for determination of heavy metals were acidified (pH < 2) and measured by inductively coupled plasma atomic emission spectrometry (ICP-AES, Optima 5300DV). Sediment sub-samples were dried by vacuum freeze-drying. Precipitates were grounded and then sieved using 200 mesh plastic sieves to remove the detrital materials before analysis.

### Analytical methods

The detail morphological characterization of sample particle size and morphology were viewed with a scanning electron microscope (SEM, Carl Zeiss Microscopy, Germany). Concentrations of SO_4_^2-^ were detected using ion chromatograph (IC, Dionex ics-1000). The BET (Brunner-Emmett-Teller) surface areas of minerals were detected by specific surface and pore size analyzer (NOVA4200E). X-ray powder diffraction (XRD) measurement with step-scanned in step intervals of 0.02°/s 2θ from 10° to 80° using 1° divergence slit was used to measure the granular crystal type, X-ray was Cu target Ka rays (λ = 0.15418 nm), the tube voltage was 40 kV, and the tube current 40 mA. In addition, the intensity diffractogram peaks of goethite or jarosite can overlap with those of schwertmannite when a mixture of the field minerals is characterized by XRD [24, 29]. Consequently, differential X-ray diffraction (DXRD) analysis was employed in soil mineralogy research to address this issue [30]. The DXRD pattern of the portion which dissolved can be obtained from subtraction between the diffraction pattern of the treated sample which multiplied by a factor of *k* and the pattern of the untreated sample [30]. And *k* could be obtained by the Eq (1).

$$k=\frac{I_{u}\text{-}Ib_{u}}{I_{t}\text{-}Ib_{t}}$$ (1)

Where u is untreated, t is treated. I_u_ and I_t_ were represented as the internal standard intensity of untreated and treated. Ib_u_ and Ib_t_ were represented as the background intensity of untreated and treated.

For purpose of quantitative, a method of evaluation system was established by using semi-quantitative XRD. Semi-quantitative XRD analysis obtained from the “external standard” approach of “*K* value method”. On account of high dissolubility and high stability, NaCl was chosen to be the prepared standard sample. *K* is calculated from the formula (2).

$$\frac{I_{j}}{I_{s}}=K\frac{W_{j}}{W_{s}}$$ (2)

Where *K* is the correction coefficient, j is the mineral, s is the external. *W*_*j*_ and *W*_*s*_ were the mass fraction of *j* and s in a mixture. *I*_*j*_ and *I*_*s*_ were represented as the intensity of *j* and *s*. Specie mass ratio was carried out to obtain the *K* value.

### Sequential extraction procedure

#### The synthesis of minerals

Schwertmanite sample was prepared by fast synthesis method called ‘oxidative synthesis’ as described by Regenspurg and Peiffer [31]. Ferrihydrite and hematite were synthesized by the methods described by Schwertmann and Cornell [32]. Goethite was synthesized using a procedure adapted from Johnson [33]. Jarosites prepared in this work were synthesized by the methods of Drouet and Navrotsky [34]. For the sake of the customization extraction scheme, synthetic minerals (schwertmannite, jarosite, goethite and ferrihydrite) were subjected to dissolution kinetics.

#### Sequential extraction procedure of field samples

The sequential extraction scheme is based on the methods described by the literature [16, 35–36] and developed according to the results obtained from dissolution experiment of synthetic minerals. Thus, a total of seven sequential extraction procedures which were suitable for the present studied sediment samples in Dabaoshan Mine were attempted. The extraction experiments were performed by using centrifuge tubes. And the extractant volume was 20 ml except where otherwise specified. It was devised by choosing a ratio of 1:15 for sediments and extractants. After the centrifugation, the liquids are used for the determining of contents of Fe, S and heavy metals, and the solid fractions are freeze-dried for analysis. The sequence of the steps was shown in Table 1.

**Table 1:** Adapted 7-steps sequential extractions sequences developed for this study.

Sequence	Extractant	Dissolved phases in this study	References
(1) water soluble fraction	deionizied water extracted for 1 h	Secondary sulfates	6
(2) exchangeable fraction	1M MgCl_2_ extracted for 1h;	readily soluble Fe salts	6, 16, 35
(3) easily reducible	0.2 M ammonium oxalate (3 pH buffer), shaken for 16 h in darkness	Schwertmannite, ferrihydite	1, 5, 29
(4) reducible oxides	0.35 M acetic acid/ 0.2M sodium citrate buffer with 50 g/L sodium dithionite (CBD) extracted for 4 h	Jarosite, Goethite, and hematite	36
(5) relatively stable mineral	0.2 M ammonium oxalate (3 pH buffer), shaken for 8 h in water bath at 80°C	Goethite, and hematite	24
(6) pooly reactive sheet silicate	12 N HCl	siderite and ankerite	16
(7) residual	Digested by 3 ml HNO_3_, 7.5 ml HF and 2.5 ml HClO_4_	Silicates	23, 24

### Quality control and quality assurance

The recoveries of extraction methods for adapted 7-steps sequential extractions sequences 97.5 ± 4.3% (n = 3) separately. The analytical accuracy of heavy metals and SO_4_^2-^ in aquatic environment were controlled with commercial standard solutions: GBW(E) 081621 (SO_4_^2-^), GSB04-1767-2004 (Cu, Zn, Mn, Pb, Cd, Ni, Cr, Fe, As). A triplicate analysis was conducted to evaluate the precision. The relative standard deviations for heavy metals and semi-quantitative XRD were below 5%.

## Results and discussion

### The variation of heavy metals concentration of water samples in Hengshi River

**Fig 2:**
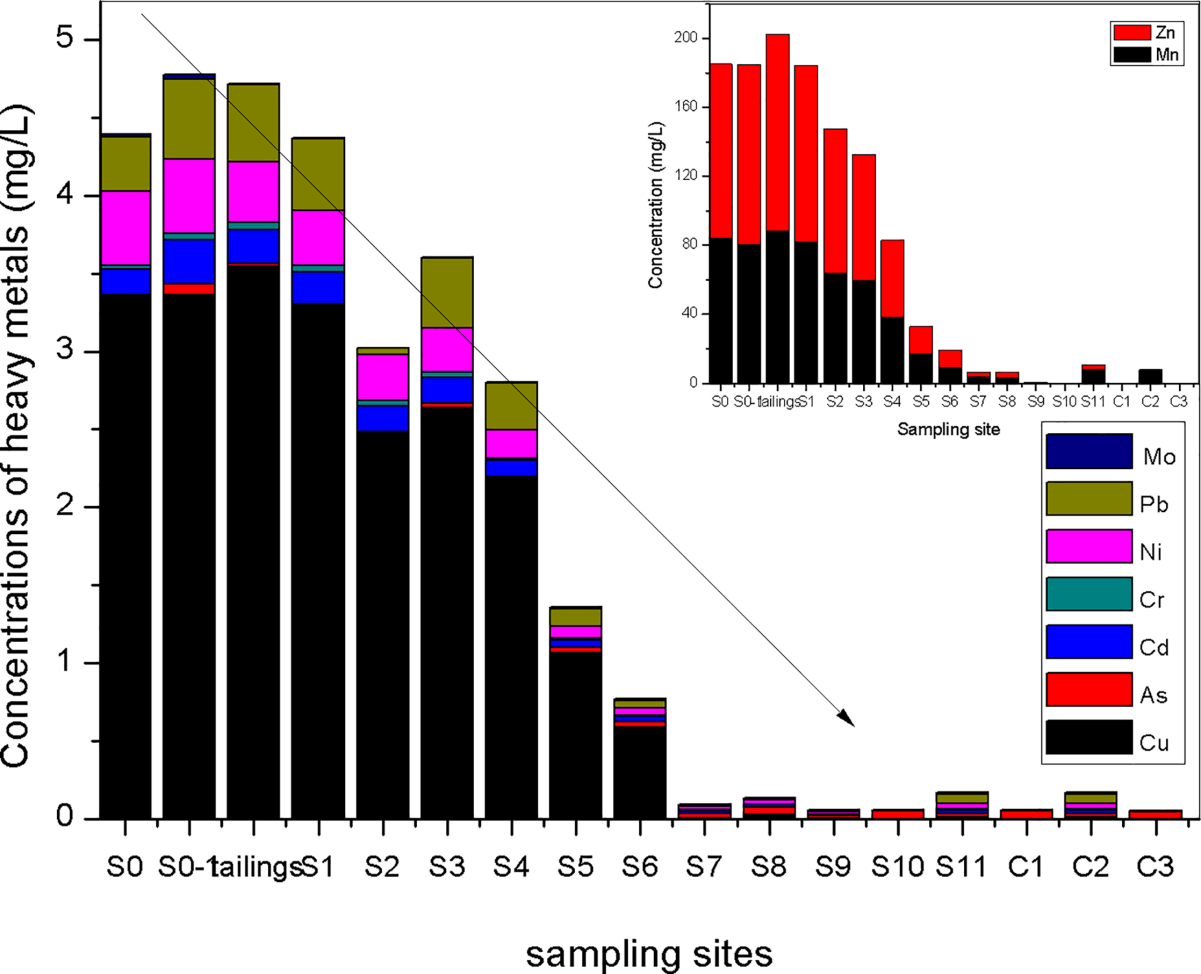
The variation of heavy metals concentration in Hengshi River.

In Fig 2, high concentrations of Zn, Mn and Cu, with low concentrations of As, Cd, Pb, Ni, and Cr were the heavy metals status in the Hengshi River. For Mn, the concentration was 83.95 mg/L at S0 but it reduced to be 7.75 mg/L at S11. In the case of S0, the concentrations of Zn, Cu, As, Cd, Pb, Cr and Ni were 101.290, 3.355, 0.024, 0.170, 0.349, 0.022 and 0.476 mg/L, respectively, while in site of S11, the concentrations decreased to be 3.164, 0.008, 0.006, 0.025,0.058, 0.006 and 0.035 mg/L. The pH values from S0 (pH = 2.50) to S11 (pH = 6.93) implied that dilution from tributaries downstream would be ~1000 times in the natural system. According to the dilution times, the concentrations of heavy metals will be ~1000 folds in S0 than those of S11. However, it is not consistent with theory values. One possible explanation for these findings is that they were caused by external loading. Another explanation is the release of heavy metals which is caused by the phase transformation of minerals.

### Calculation of geochemical model PHREEQC

**Fig 3:**
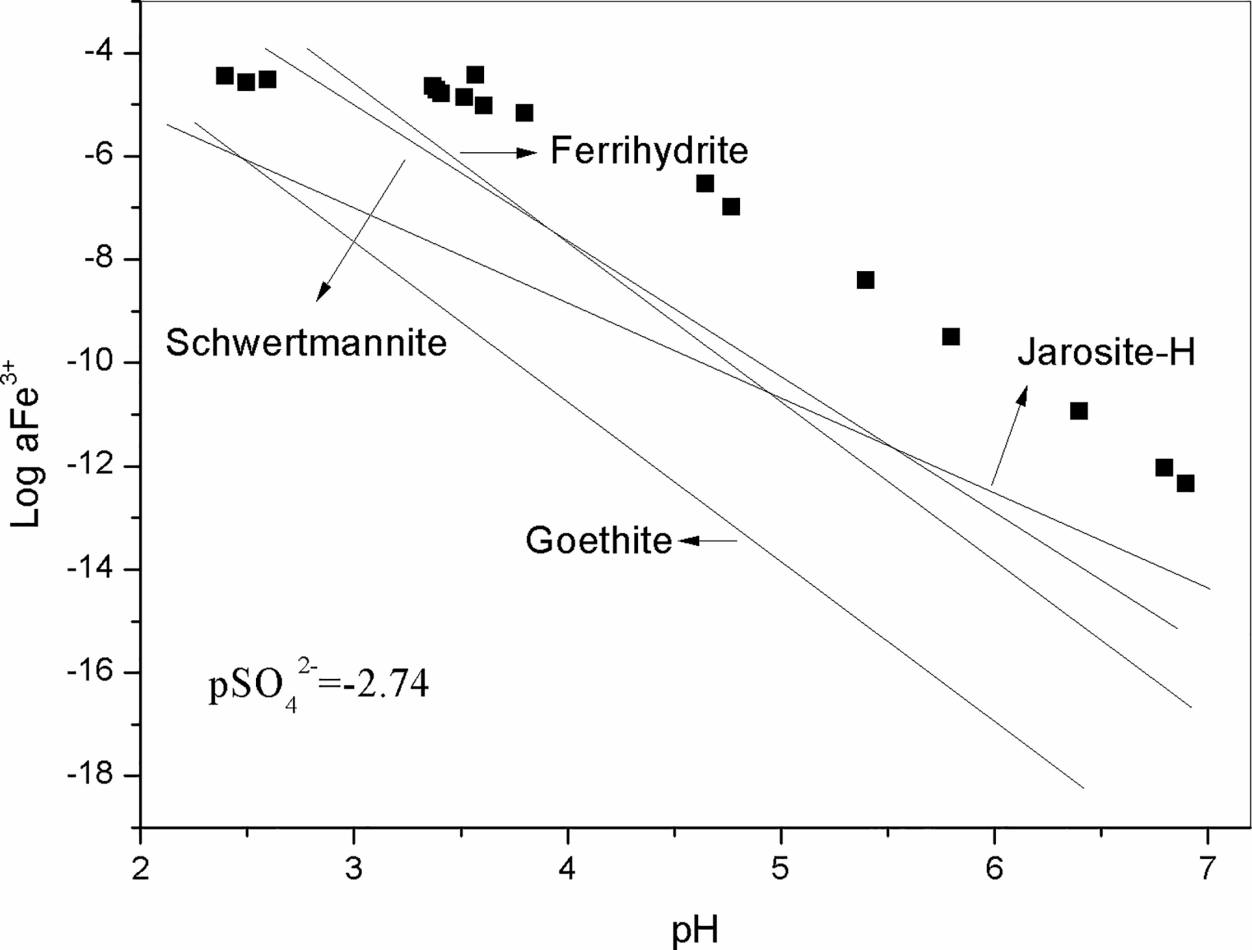
Activity of Fe^3+^ relative to pH (Solubility lines are as follows: goethite (log aFe^3+^ = 1.4-3pH), ferrihydrite (logaFe^3+^ = 4.3-3pH), and schwertmannite (log aFe^3+^ = 2.83–2.6pH), jarosite (log aFe^3+^ = -2.5–1.67 pH)).

The geochemical model PHREEQC is widely used for modeling of non-equilibrium mineral dissolution and precipitation to predict mine drainage quality [37]. Saturation Index (SI) is applied for the research about saturation degree of particular minerals. According to the field measured pH and the concentration of Fe^3+^ determined in the laboratory, the mineral SI calculations were simulated by PHREEQC software in the present study. As shown in Fig 3, it indicated that the minerals (e.g., schwertmannite (SI > 25), goethite (SI > 4), jarosite (SI > 2) and ferrihydrite (SI >17)) were possible to be precipitated in the study area. As suggested by the literature [5–6], Owing to the high reactivity and large specific area of hydrous oxides, Fe oxyhydroxide minerals are likely to adsorb or sequester heavy metals. So the mobility, bioavailability and toxicity of heavy metals are largely controlled by chemical reactions which take place at the hydrous oxide/water interface.

### Extractability trends of synthetic minerals

Synthetic schwertmannite, ferrihydrite, jarosite, and goethite were used for extraction studies. As can be seen from Table 2, schwertmannite (100%) and ferrihydrite (100%) can be dissolved by 0.2 M ammonium oxalate in 16 h in darkness. 0.35 M acetic acid and 0.2 M sodium citrate buffer with 50 g/L sodium dithionite extracted for 4 h were known as CBD, a strong reducing agent which is commonly used for remove crystalline iron oxide fraction. Results showed that CBD can dissolve100% jarosite and larger quantities of goethite (72.5%) and hematite (62.0%). Eventually, 0.2 M ammonium oxalate (3 pH buffer), shaken for 4 h in water bath at 80°C was chosen for highly crystalline iron oxide minerals. And it is suitable for hematite extraction (38.0%) for 24 h, with 27.5% extraction efficiency for goethite. Hence, the results of this extractability study of synthetic minerals contributed to extraction of field samples. And the recovery of Fe varied between 92.1% and 103.2% for the extraction.

**Table 2:** Percentage of iron removed from synthetic minerals in each step of adapted 7-steps sequential extractions.

items	schwertmannite	ferrihydrite	jarosite	hematite	goethite
DI water	0	0	0	0	0
MgCl_2_	0	0	0	0	0
NH_4_-oxalate	100%	100%	0	0	0
CBD	0	0	100%	62%	72.5%
NH_4_-oxalate at 80°C	0	0	0	38%	27.5%
HCl	0	0	0	0	0
HNO_3_, HF and HClO_4_	0	0	0	0	0

### Solid-phase minerals in the sediments

#### Mineralogical characterization

**Fig 4:**
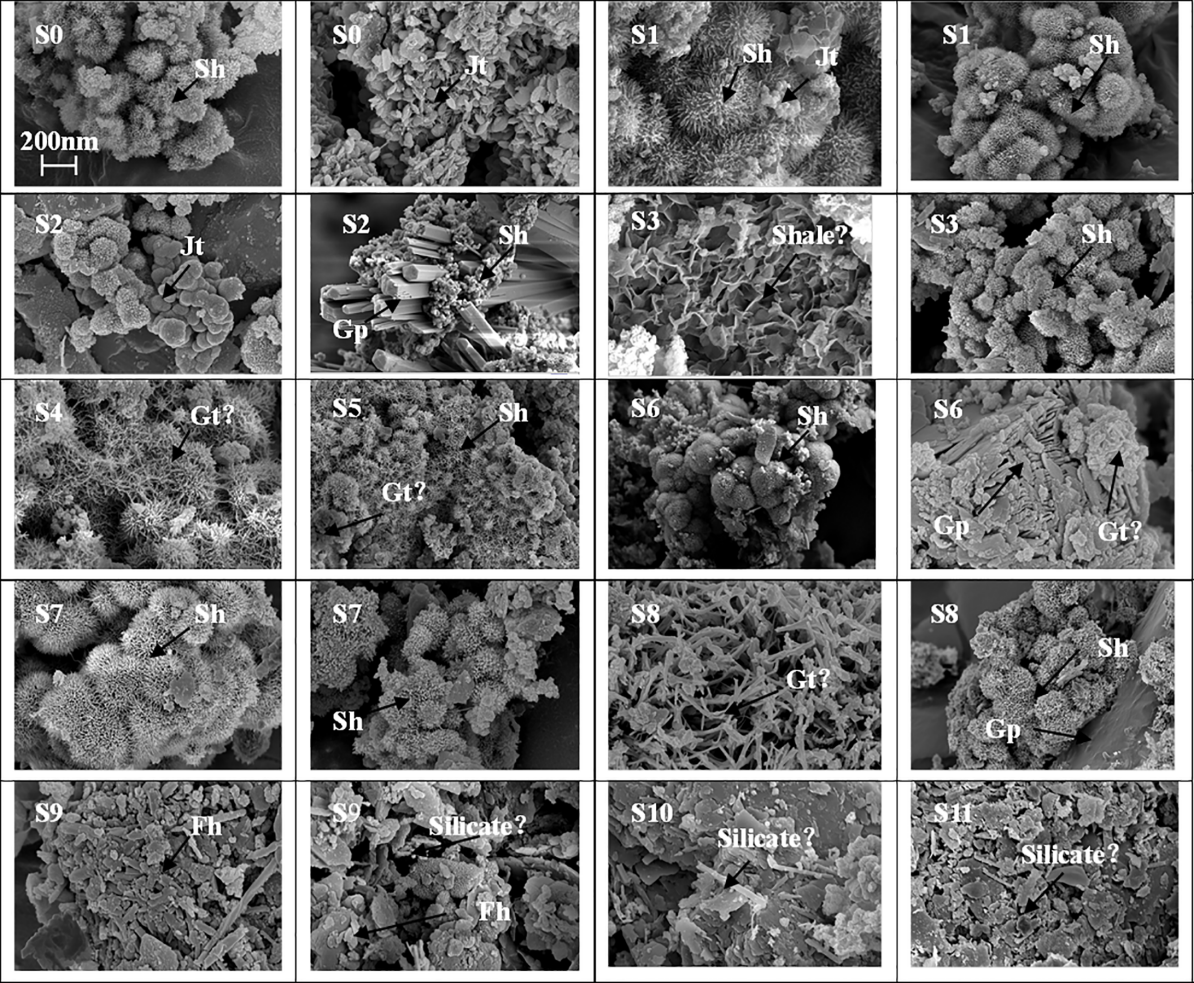
SEM images of sediment samples before extraction (Sh: schwertmannite; Jt: jarosite; Gt: goethite; Fh: ferrihydrite; Gp: gypsum; (The scale of all figures is the same)).

As shown in Fig 4, the “pincushion” morphology has been observed in samples of S0 to S8 which is in loose precipitates; it revealed that schwertmannite may be found in sites of S0 to S8 [38]. But it will be prone to be schistose then needle-like when pH increased. Particles or crystal aggregates have spheroids with a diameter of ~ 200 nm to 2 μm. The “needle” or “discrete acicular crystals” morphology was similar to that previously described in literature [32–33] for goethite in S4 to S8. Compared to the synthetic goethite, the natural goethite was hollow inside, owing to the transformation of schwertmannite into goethite spontaneously in AMD environment [1, 4]. For the sample of S0, S1 and S2, hexagonal and plate-like morphology for the jarosite were observed. And lath-like crystals refer to gypsum [39], owing to the dissolution of limestone and the high concentration of SO_4_^2-^ in the AMD. Ferrihydrite typically forms rounded coalesced aggregates mainly presents as networks [36], which was found in the samples of S9, S10 and S11. Another morphology liked layered and lamellar structure were speculated to be sillcates, shale or mica in all samples. In natural systems, due to the transformation of poorly crystalline minerals, natural settings are represented by many types of minerals.

#### Semi-quantification of minerals in sediments

**Fig 5:**
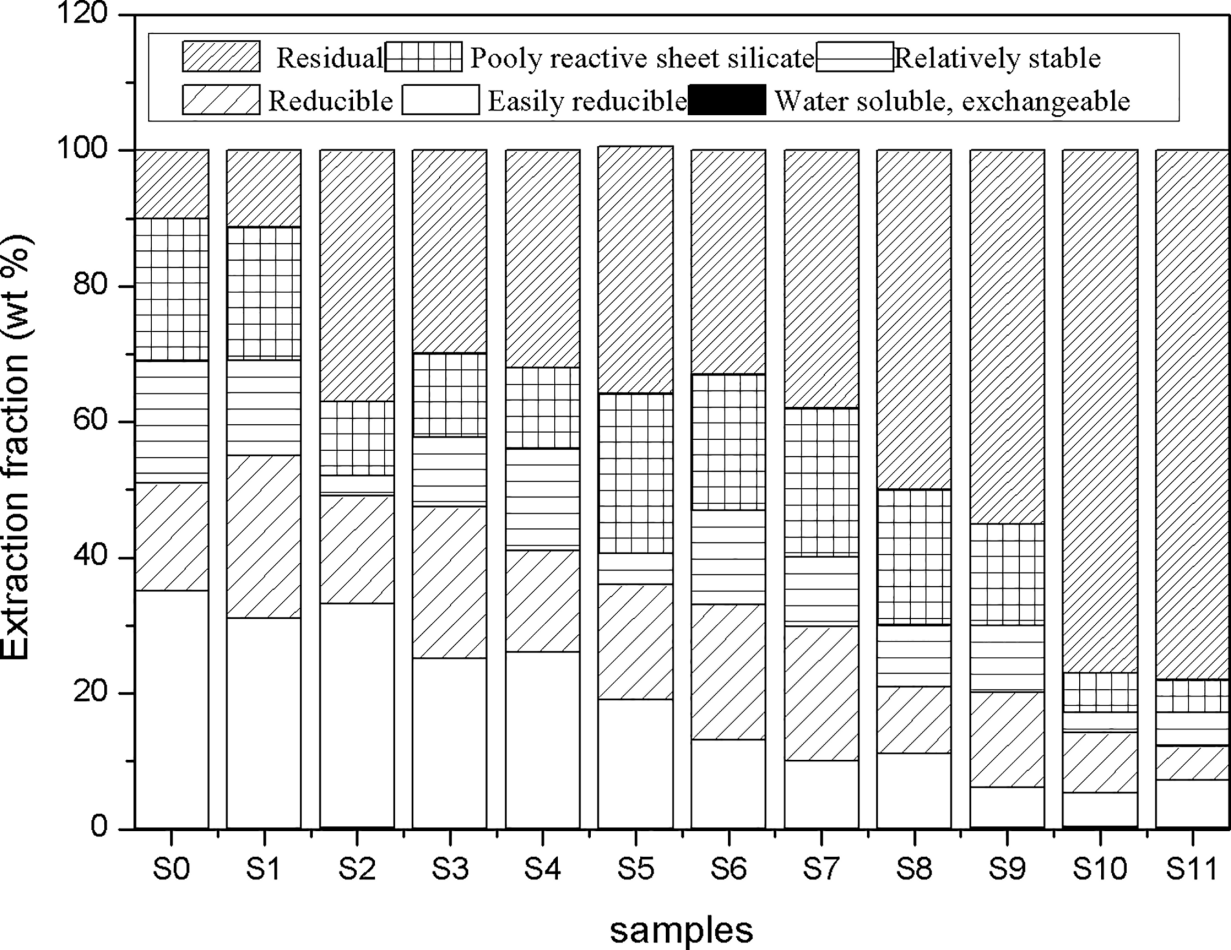
Sequential extractions of sediment samples from sampling sites (S0-S11).

Based on results of extractability trends of synthetic minerals, all the samples were subjected to the sequential extractions (Table 1) step by step. As shown in Fig 5, compared to other fractions, a little variation in quality of samples (0.1 ~ 0.3 wt%) after extraction by deionizied water and MgCl_2_ referred to secondary sulfates and readily soluble Fe salts.

**Fig 6:**
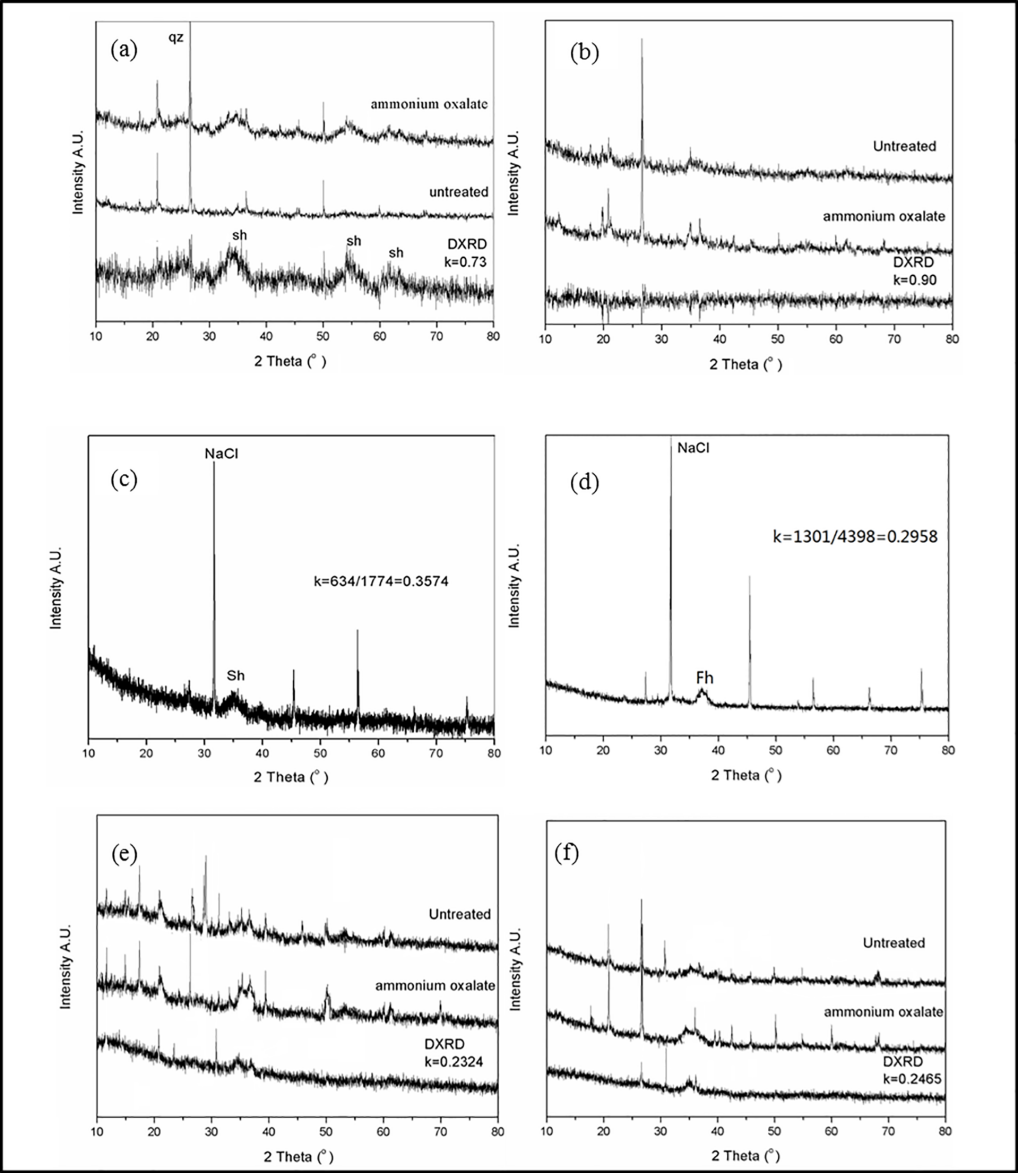
DXRD and semi-quantitative XRD patterns of sediment samples. ((a) and (b) DXRD patterns refer to samples S0 and S1; (c) and (d) refer to the mixture of NaCl with Sh and Fh; (e) and (f): Semi-quantitative XRD patterns of samples S7 and S8; the Sh: schwertmannite; Fh: Ferrihydrite).

A survey of the literature available shows that schwertmannite and ferrihydite are meta-stable and commonly associated with goethite [5]. In order to discriminate them in step 3, DXRD were used to facilitate the detection between schwertmannite and ferrihydrite [5]. Prior to the results of XRD (S1 Fig), samples were mostly mixed phases composed of various minerals including quartz. Therefore, SiO_2_ was used as internal standard substance. And k is an intensity correction factor which can be calculated from the formula (1). Take an example of S0, DXRD pattern was obtain by the diffraction pattern of the treated sample subtracted from the diffraction pattern of the untreated sample by a factor of 0.73 (Fig 6(A) and 6(B)), The ratio of Fe/S of leaching extraction in samples of S0 was 5.76, the average chemical formula of schwertmannite obtained from Step 3 was Fe_8_O_8_(OH)_5.22_(SO_4_)_1.39_. Nevertheless, for the samples of S7 and S8, DXRD pattern contained significant amounts of schwertmannite and a small amount of ferrihydrite. As shown in Fig 6(C) and 6(D), the *K* values of schwertmannite and ferrihydrite which calculated by Eq (2) were 0.3575 and 0.2383, respectively. So the relative weight ratio of schwertmannite in sample S7 and S8 approximately evaluated by semi-quantitative XRD analysis is 7.8 wt % and 6.5 wt%.

The step 4 is proposed for the speciation of solubilized reducible oxides like jarosite, goethite and hematite. Based on literature, schwertmannite forms at the pH of 2.8–4.0, followed by the goethite in the downstream as the pH rising to 5.0–6.9. Nevertheless, a majority of goethite may be derived from the transformation of schwertmannite or ferrihydrite. In addition, hematite also could be detected in the fractions. This may be explained by hematite was generally formed by dehydration of ferrihydrite or goethite [36]. Sample S1 showed the maximum contents 24 wt % of reducible oxides in all the samples, followed by S3 (22.4 wt %), and S11 was the minimum (5.0 wt %).

As obscured in the Fig 5, the results suggest that this extraction may give an indication of goethite and hematite present in the sediments. The fraction of relatively stable minerals decreased with the distance of the AMD source, except for the sites of S2 and S5. S0 received the maximum content of 18.1 wt %. For step 6, the poorly reactive sheet silicate was believed to be abundance being in the sediments. In the sample from S0 to S9, the contents of poorly reactive sheet silicate ranged from 10.9 wt % to 23.5 wt %.

Finally, for the residual, samples were submitted to the last step which was digested by mixed acids. Most of the residual were proved to be the quartz and phyllosilicates. As shown in the Fig 5, the samples of S0 and S1 have the less proportion of 10.0 wt% and 11.3 wt%. From S2 to S7, the variation of residual was similar to that of each, and it ranges from 30.0 wt% to 38.1 wt%. But interestingly, with respect to S8, the higher content of residual (50.0 wt %) than that of S0-S7 may be attributed to the human activity in Shangba Village. For samples of S9, S10 and S11, results showed that the higher pH values were measured in the Hengshi River, with corresponding to a high content of residue (55.1 wt%, 77.0 wt% and 78.0 wt %).

### The content of heavy metals in the precipitates

**Fig 7:**
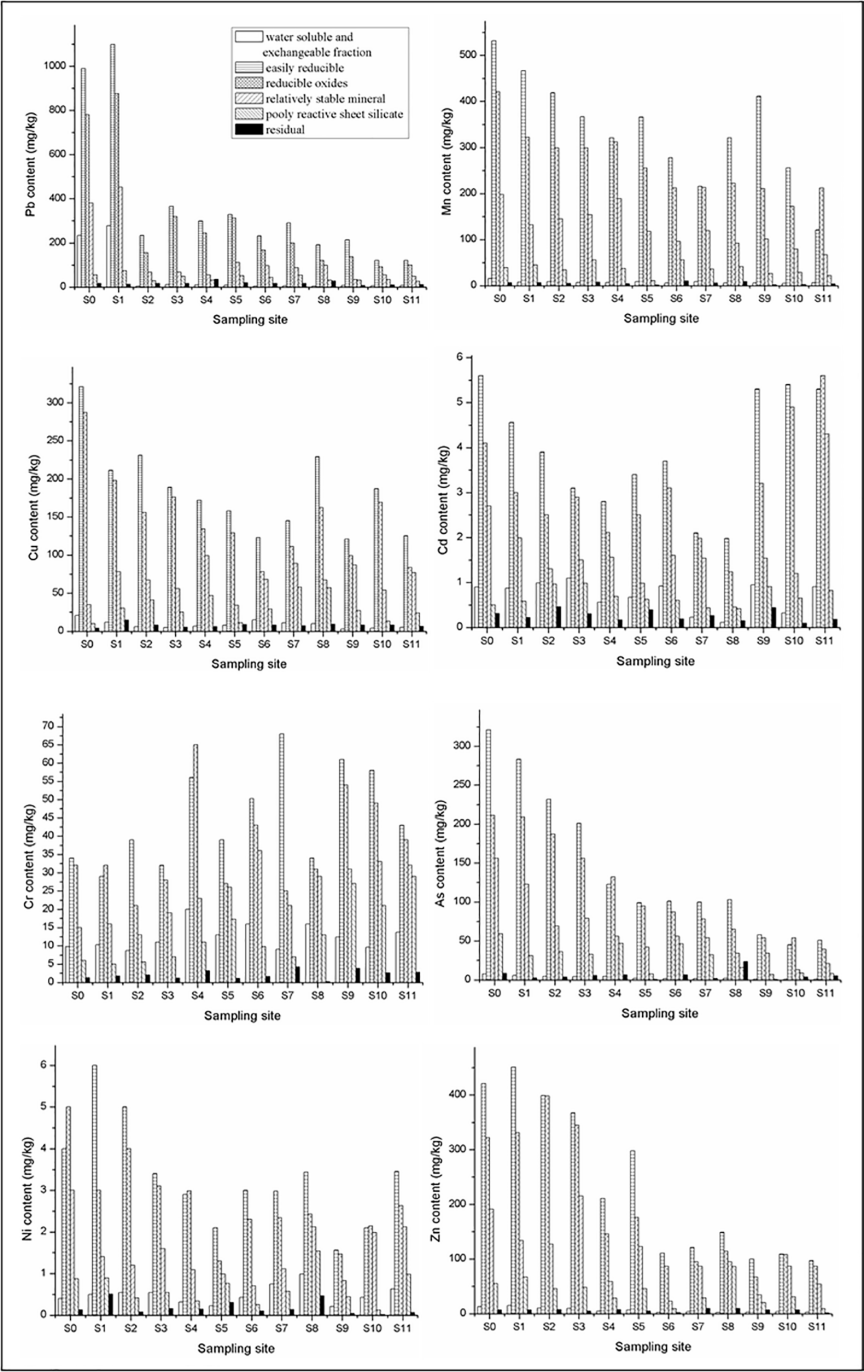
The content of heavy metals in the precipitates (A triplicate analysis was performed and the relative standard deviations were below 5%).

Based on the results above, jarosite, schwertmannite, goethite and ferrihydrite alternately appeared in the sediments with the increasing pH from 2.50 to 6.93 along the Hengshi River, as well as the heavy metals. The evolution of trace element concentrations in precipitates showed different patterns. Soluble and exchangeable fractions of metals were bound to the mineral surface and they are more easily to release into the aqueous environments. As shown in Fig 7, schwertmannite (easily reducible fractions in step 3) has higher enrichment for Pb (1099.00–191.00 mg/kg), Cu (321.00–123.00 mg/kg), Cr (56.00–29.00 mg/kg), Zn (451.00–111.00 mg/kg), and Mn (532.00–211.00 mg/kg) and As (322.00–99.80mg/kg) at S0 to S8 obviously higher than those at other sites. But Ni (11.23–4.55 mg/kg) and Cd (14.11–4.34mg/kg) had the lower order affinity in all samples. In the cases of Pb, Zn and Mn, of which the concentrations were relative high (Pb > 2000 mg/kg, Zn and Mn > 1000mg/kg) at S0 but dropped progressively to values close to 314.00 mg/kg, 251.00 mg/kg, 432.00 mg/kg at the site of S11. The decreases may be related to the increase of pH along the river and the adsorption on mineral surface and co-precipitation in the mineral structure [40]. By contrast, the lower contents of As, Cu and Cr in step 4 and 5 suggesting that goethite and ferrihydrite are less efficient sink for As, Cu and Cr than schwertmannite and jarosite. Compared to schwertmannite, reducible oxides like goethite and higher order ferrihydrite have lower affinity with heavy metals leading from a decreasing in surface area [41, 42]. The specific surface area of Fe-oxyhydroxide precipitates in the study area was measured to vary between 23.00 and 198.00 m^2^/g. In sites of S9, S10 and S11, relatively low values of heavy metals were detected in the sediments, corresponding to the low heavy metals adsorption affinity of minerals found in these locations. It should be noted that the decreasing metal contents in the stream from S0 to S11 suggested that Fe oxyhydroxide minerals played a vital role in the fate and transport of heavy metals in AMD system. Just as reported by Acero et al. [38] and Fritzsche et al. [43] elements removed from solution must be retained in the solid phase throughout their transformation into other mineral phases. Besides, high SO_4_^2-^ concentration (ranged from 2021.00 mg/L to 82.00 mg/L located from S0 to S11) in AMD will be beneficial to the formation of schwertmannite or jarosite which have high affinity of heavy metals. So SO_4_^2-^ concentrations will be another possible influencing factor. Moreover, SO_4_^2-^ ligands with related to heavy metals and the negative charge will enhance the adsorption of metals when SO_4_^2-^ adsorption on the surface of minerals [41–42, 44].

### The enrichment of heavy metals in minerals

**Fig 8:**
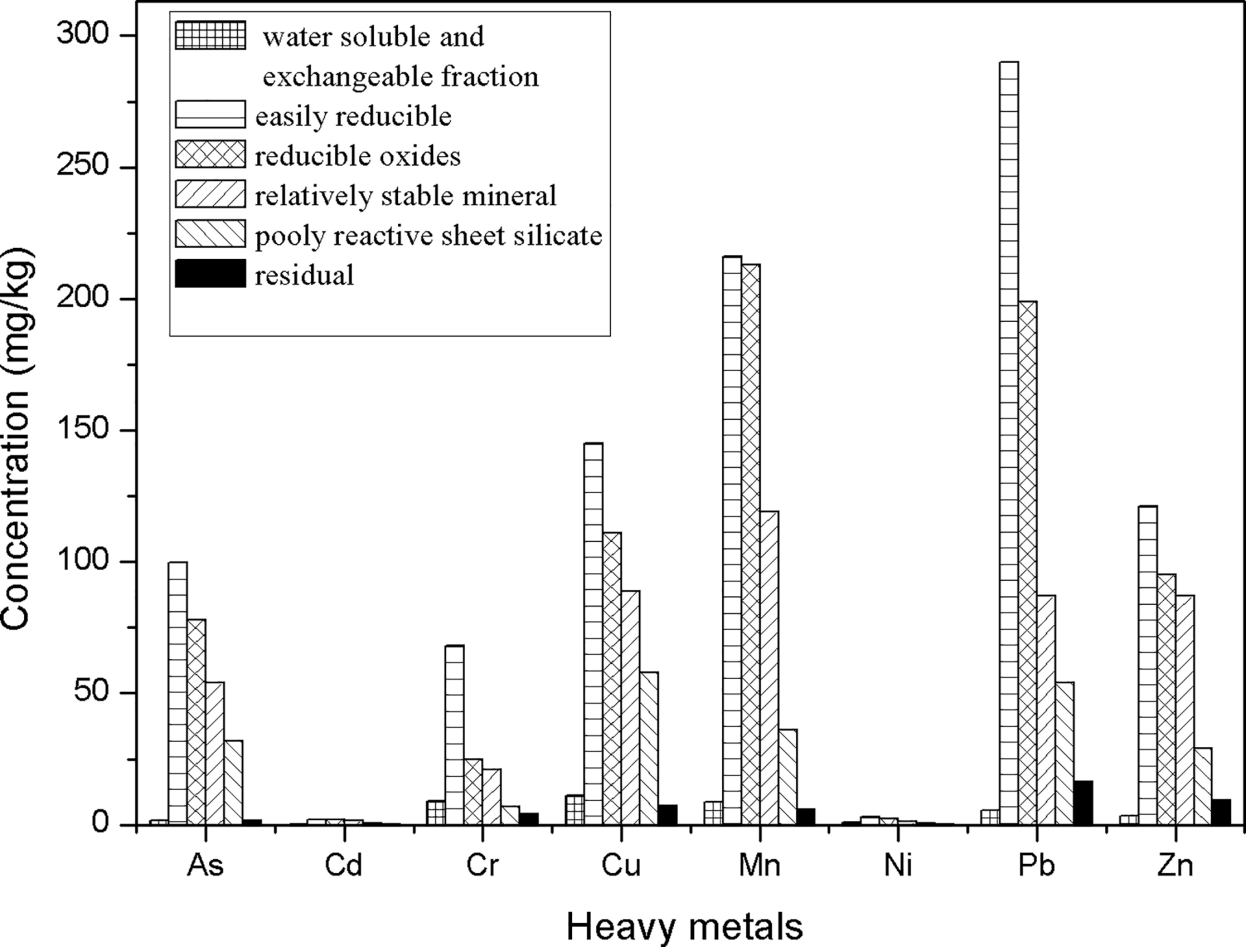
Comparison of heavy metals contents in each extract phase of sample S7 (A triplicate analysis was performed and the relative standard deviations were below 5%).

In order to compare the sorption affinity varying between precipitates, S7 was taken as the typical sample for the experiments, owing to the variety of minerals in S7. As discussed above, the precipitates obtained in this current study were involved of schwertamnite, jarosite, goethite, ferrihydrite and silicates, etc. And results of Fig 8 suggested that about 82.07–91.21% heavy metals were determined in the fractions of easily reducible, reducible oxides and relatively stable mineral which attributed to dominant minerals like schwertamnite, jarosite, goethite and ferrihydrite. The occurrence of these minerals were consistent with the previous reports that trace metals associated with minerals is adsorbed either on the surface or inside tunnels according to Burgos et al. [45]. Moreover, according to Table 3, the clear positive correlation coefficients between heavy metals and Fe indicate the dominated migration of heavy metals associated with the co-precipitation of secondary minerals. As shown in Fig 8, the enrichment of heavy metals in easily reducible was the highest in the extract phases, followed by reducible oxides and relatively stable mineral. That is to say, the species of minerals take a great effect on the attenuation of trace metals [38, 46]. And the contents of Pb, As, Zn, Mn, Cu, Cr, Ni and Cd in easily reducible (schwertmannite) were 290.00, 99.80, 121.12, 216.10, 145.00, 68.01, 2.98 and 2.11 mg/kg, respectively. Thus, the qualitative retention scale can be included as Pb > Mn > Zn > As ≈ Cu > Cr > Cd ≈ Ni. And Fe-minerals are the critical factors in controlling the mobility of heavy metals along a river watershed affected by acid mine drainage in Dabaoshan Mine.

**Table 3:** Correlation coefficients calculated for the elements in the sediment samples.

	Fe	Mn	Cu	Zn	As	Cd	Cr	Ni	Pb
Fe	1	-	-	-	-	-	-	-	-
Mn	0.854**	1	-	-	-	-	-	-	-
Cu	0.907**	0.632**	1	-	-	-	-	-	-
Zn	0.903**	0.927**	0.508*	1	-	-	-	-	-
As	0.882*	-0.122	0.361	-0.079	1	-	-	-	-
Cd	0.842**	0.793**	0.662**	0.686**	0.298	1	-	-	-
Cr	0.982*	0.933**	0.827**	0.864**	0.088	0.616**	1	-	-
Ni	0.989*	0.952**	0.779**	0.829**	0.066	0.912**	0.635**	1	-
Pb	0.618**	0.905**	0.520*	0.853**	-0.061	0.676**	0.530**	0.843**	1

** means the correlation with p value > 0.01

* means the correlation with p value > 0.05

## Conclusions

The developed sequential extraction procedure combined with DXRD and semi-quantitative XRD in the current study provide greater information regarding relationship between minerals and heavy metals in sediments associated with AMD. Results showed that mainly components jarosite, schwertmannite, goethite and ferrihydrite alternately appeared along the Hengshi River. The easily reducible fraction exerts higher affinity of metals while compared with reducible oxides and relatively stable minerals. A qualitative retention scale Pb > Mn > Zn > As ≈ Cu > Cr > Cd ≈ Ni was proposed. Fe-minerals are the critical factor in controlling the mobility of heavy metals in Dabaoshan Mine. Finally, our data suggested that massive immobilization of potentially toxic metals in the precipitates will release into river leading to cause adverse impacts on stream ecosystem. Thus, attention must be paid for the precipitates which control the fate and transport of trace metals when designing for remediation strategies.

## Supporting information

S1 Fig XRD patterns of sediment samples. (DOC)
