# Supplementary material for: Mineralogical characteristics of sediments and heavy metal mobilization along a river watershed affected by acid mine drainage
Source: PLoS One. 2018 Jan 5;13(1):e0190010. doi: 10.1371/journal.pone.0190010 (PMC5755774; doi:10.1371/journal.pone.0190010)
Supplement: S1 Fig — (DOC) [file pone.0190010.s001.doc]

**Mineralogical characteristics of sediments and heavy metal mobilization along a river watershed affected by acid mine drainage** Yingying Xie^1^, Guining Lu^1,2,3*^, Chengfang Yang^1^, Lu Qu^1^, Meiqin Chen^1,4^, Chuling Guo^1,2^, Zhi Dang^1,2,3*^

1. School of Environment and Energy, South China University of Technology, Guangzhou 510006, China
2. The Key Laboratory of Pollution Control and Ecosystem Restoration in Industry Clusters, Ministry of Education, South China University of Technology, Guangzhou 510006, China
3. Guangdong Provincial Engineering and Technology Research Center for Environmental Risk Prevention and Emergency Disposal, South China University of Technology, Guangzhou 510006, China
4. School of Environmental and Biological Engineering, Guangdong University of Petrochemical Technology, Maoming 525000, China

Corresponding authors
E-mail: GNLu@foxmail.com (G. Lu), chzdang@hotmail.com (Z. Dang)


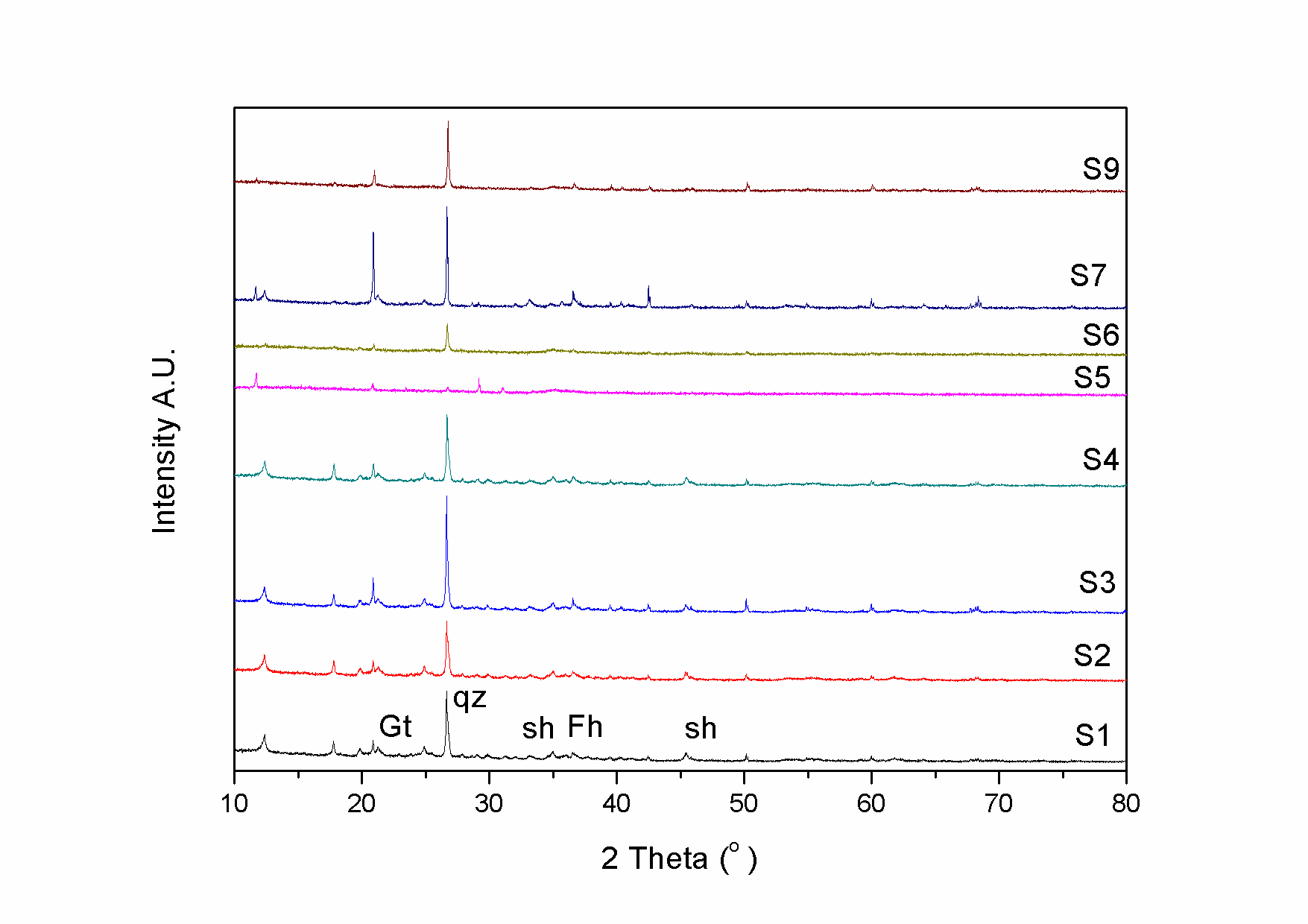


S1 Fig. XRD patterns of sediment samples
